# Juvenile Granulosa Cell Tumor of the Testis: Prenatal Diagnosis and Management

**DOI:** 10.1055/s-0039-3400275

**Published:** 2019-12-13

**Authors:** Fabrizio Vatta, Alessandro Raffaele, Noemi Pasqua, Stefania Cesari, Piero Romano, Gian Battista Parigi, Luigi Avolio

**Affiliations:** 1 Department of Pediatric Surgery, Fondazione IRCCS Policlinico San Matteo and Università degli Studi di Pavia, Pavia, Italy; 2 Department of Pathology, Fondazione IRCCS Policlinico San Matteo and Università degli Stud di Pavia, Pavia, Italy

**Keywords:** prenatal ultrasound, juvenile granulosa cell tumor, testicular tumors

## Abstract

Prepubertal primary testicular tumors account for ∼1% of all pediatric solid tumors. We report a new case of prenatal diagnosis of juvenile-type granulosa cell tumor (JGCT). A fetal ultrasound performed at the 38th week of gestation for suspected nonvertex presentation identified a left multilocular septated cystic testicular mass, suggestive for JGCT. At birth, a painless left scrotal mass was detected. Ultrasound re-evaluation excluded torsion of the testis. Tumor markers and abdominal ultrasound were normal for age. Inguinal exploration revealed a cystic mass beneath the tunica albuginea that had replaced all the normal parenchyma. Since organ-sparing surgery was thus not feasible, an orchiectomy was performed and diagnosis of JGCT was confirmed. At 7-year follow-up, the child presented an uneventful outcome. Our case shows that neonatal JGCT, which has an intrauterine genesis, can be diagnosed prenatally by ultrasound in the last weeks of pregnancy.

## Introduction

Juvenile-type granulosa cell tumor (JGCT) of the testis, although rare, is the most common testicular neoplasm in the first 6 months of life. 1 It is frequently diagnosed in the neonatal period; it is uncommon in older children and exceptional in adults. 2 Typical presentation is a painless neonatal scrotal mass 3 ; occasionally it occurs in cryptorchidic testes 3 4 5 or in infants with abnormal karyotypes and ambiguous genitalia 3 ; all cases reported have had a benign outcome. 3 6 Inguinal orchiectomy was invariably considered the treatment of choice but new treatment trends advocate a trans-scrotal approach 7 and testis-sparing surgery where preoperative staging determines that this is safe. 6 8 We report a case of JGCT of the testis prenatally diagnosed, an event described only twice in the literature so far, 7 9 followed by inguinal orchiectomy.

## Case Report

A healthy 2-day-old newborn was admitted to our department for a left scrotal mass. He had undergone a prenatal ultrasound a week before delivery (38th week of gestation) for suspected nonvertex presentation. On that occasion, a left testicular cystic mass (2 × 2 cm), suspected to be a JGCT due to its multicystic aspect, was identified. At clinical presentation, the left testicle appeared in situ, with increased size and consistency, while the right testicle and the penis were normal.

Ultrasound revaluation after birth excluded torsion of the testis and confirmed the presence of a voluminous multicystic left testicular mass without normal-appearing parenchyma. Serum α-fetoprotein (AFP) and β-human chorionic gonadotropin (β-HCG) were normal for age. Karyotype was normal. Abdominal ultrasound did not show any anomalies. Following surgical oncological criteria, we opted for an inguinal approach: after groin incision, the spermatic chord was identified and clamped at the level of the deep inguinal orifice. Testis examination revealed a cystic mass beneath the tunica albuginea replacing all normal parenchyma. We performed funiculo-orchiectomy, since organ-sparing excision of the mass was considered not possible. There was no evidence of enlarged inguinal lymph nodes.

**Fig. 1:**
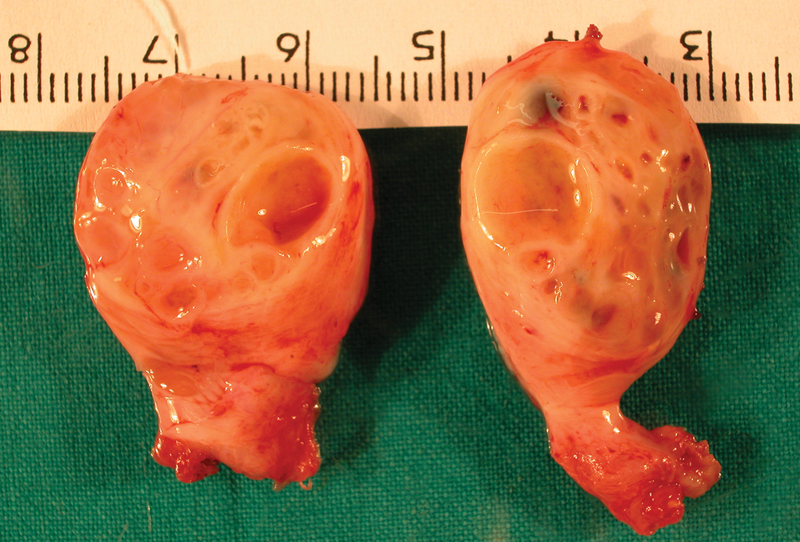
Juvenile granulosa cell tumor gross specimen showing cystic mass replacing all normal testicular parenchyma.

Gross examination of the surgical specimen revealed a well-circumscribed 2 × 1.5 cm white mass. The cut surface showed multiple, thin-walled cysts containing clear fluid ( Fig. 1 ).

**Fig. 2:**
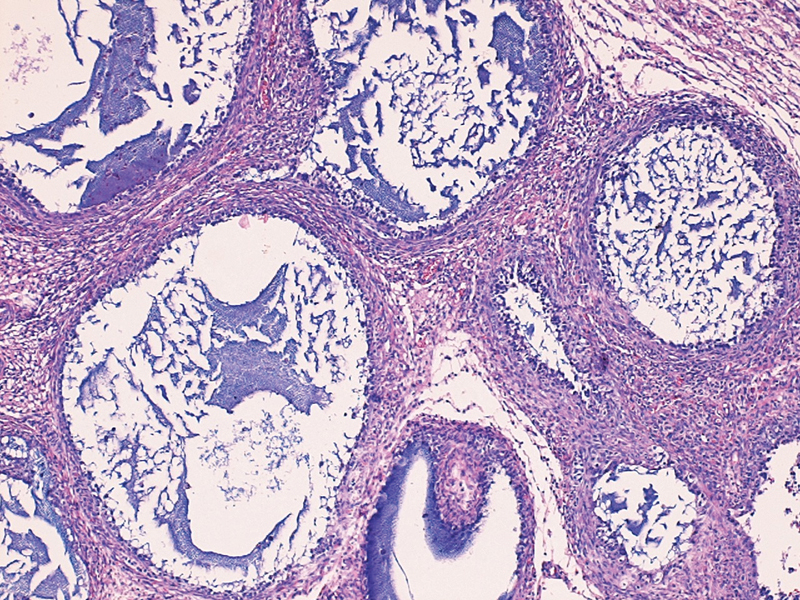
Multiple variably sized follicles containing basophilic material and lined by one to several layers of cells with pale cytoplasm.

Microscopic examination showed multiple follicle-like structures of varying size, round to oval, filled with basophilic fluid stained by mucicarmine. The follicles were lined by variable layers of cells with round hyperchromatic nuclei and scant pale cytoplasm ( Fig. 2 ). Nucleoli were not prominent but were occasionally visible, and nuclear grooves were absent. Mitotic activity was low. The stroma was composed of edematous fibrovascular tissue that formed a dense layer of spindle cells around the follicles. The neoplastic cells were immunoreactive for α-inhibin, CD99, and calretinin antibodies. Focal expression of cytokeratin was also observed. Immunoreactions for FLAP, AFP, CD30, vimentin, and β-HCG were negative. Immunostaining for Ki67 showed a low proliferative index (1% of the tumor cells).

Based on morphologic and immunohistochemical findings, a diagnosis of JGCT was formulated. The tumor did not extend into the spermatic cord, epididymis, or tunica vaginalis. Nevertheless, only a small rim of residual testis was present.

The baby was discharged 2 days after the surgical procedure. Seven-year-follow-up was uneventful.

## Discussion

Prepubertal primary testicular tumors account for ∼1% of all solid pediatric tumors. Gonadal stromal tumors, including Leydig cell, Sertoli cell, and granulosa cell tumors, account for ∼8% of these neoplasms and are therefore extremely rare. JGCT accounts for only 1.2% of all prepubertal testis tumors recorded in the Pre-pubertal Testis Tumor Registry. 10 Nevertheless, JGCT is the most common stromal cord neoplasm of the testis in the first 6 months of life. 3 This tumor may be associated with anomalies of the genitalia or sexual chromosome abnormalities. JGCT is considered a benign tumor since no reports of metastatic disease are described in literature. 3 6

Differential diagnosis for JGCT includes evaluation for yolk-sac and other sex cord-stromal tumors. 3 11 The juvenile form can be distinguished from the adult one by the lack of nuclear grooves and Call–Exner bodies, and the greater degree of irregularity in size and shape of the follicles, which show intraluminal basophilic fluid.

Overall, the typically very young age of patients is helpful in diagnosis 11 12 : yolk-sac tumor, also relatively frequent in infants, is usually seen beyond 6 months of age. 3 The presence of follicular structures, the absence of the various characteristic patterns of yolk-sac tumor, a lack of reactivity for AFP, and positivity for α-inhibin favor a diagnosis of JGCT. 6 12 13 Sertoli cell tumors usually exhibit a prominent tubular differentiation with bland cytologic features and little mitotic activity, in contrast with the follicular pattern and more immature-appearing nuclei of JGCT. Furthermore, in the largest series of Sertoli cell tumors, no patient was under 15 years of age. 11 12 13 14

In one case reported in the literature, retrospectively reviewed prenatal ultrasounds of a newborn treated for JGCT showed a testicular mass. 15 To our knowledge, we report only the third case where JGCT was suspected prenatally. 7 9 15 As in our experience, prenatal diagnosis in the two previously reported cases was accidental and made at 36 and 38 weeks of gestation, respectively. In our case, the mass was not observed at the previous routine ultrasound examination. So, suggesting that in utero diagnosis of this rare tumor is possible only in the last weeks of pregnancy.

Due to their benignancy, JGCTs have been treated with testis-sparing surgery 6 8 16 where it is possible to identify a rim of normal parenchyma; in these cases, normal preoperative levels of AFP and intraoperative biopsies and frozen section analysis are essential to exclude malignant lesions. 6 Likewise, the less invasive trans-scrotal approach used in a case reported in 2012 may be safe and feasible where AFP levels are normal and where ultrasound strongly suggests the presence of a JGCT, 7 although the oncological outcome for this patient is unknown due to lack of long-term follow-up. However, if this procedure is not possible due to a tumor completely replacing the testis as in our case, orchiectomy is the only treatment option.

## Conclusion

Our case shows that JGCT can be diagnosed prenatally in the last weeks of gestation. In our case, diagnosis in the 38th weeks of pregnancy after unremarkable previous routine ultrasounds indicates late fetal development of the tumor. Although successful trans-scrotal removal of JGCT has been reported, inguinal orchiectomy remains the safest way in terms of oncological criteria.

## Notes

New Insights and the Importance for the Pediatric Surgeon Juvenile granulosa cell tumor is a benign tumor of the testis that should be treated, when possible, with a conservative approach.
